# Right ventricular responses to abnormal preload and afterload: a comparison of right ventricular regional displacement by cardiac MRI to elevated right heart pressures by catheterization

**DOI:** 10.1186/1532-429X-14-S1-P89

**Published:** 2012-02-01

**Authors:** Nabil A Shafi, Jeannette McLaughlin, Peter D Rhee, Michael Passick, George A Petrossian, Madhavi Kadiyala, Nathaniel Reichek, Jie J Cao

**Affiliations:** 1 Department of Research and Education, St. Francis Hospital- The Heart Center, Roslyn, NY, USA; 2 Division of Cardiology and Department of Biomedical Engineering, SUNY- Stony Brook University, Stony Brook, NY, USA

## Background

Cardiac MRI (CMR) is valuable in assessing right ventricular (RV) structure and function. However, RV mechanical parameters such as regional wall displacement have not been fully explored using CMR. We sought to understand effects of increased RV preload and afterload on RV regional wall displacement.

## Methods

Research subjects included 10 normal volunteers undergoing CMR and 36 patients undergoing clinically indicated right and left heart catheterization and a research CMR within 5 hours. Cine images were acquired to evaluate RV volumes and RVEF. Radial and longitudinal RV segmental displacement during systole were evaluated at the right sided septal endocardium and RV free wall on 4-chamber cine images using Feature Tracking software. Hemodynamics assessed during catheterization included RVSP, RVEDP, PASP, mean PAP and LVEDP. In normal controls, RVSP was estimated by echocardiography.

## Results

Mean age was 60 years in patients and 55 in controls (NS). In the patient group mean RV end diastolic volume was 73±41 ml/m2 and RVEF 50± 15%. Reduced radial displacement of the septum was significantly associated with increased RVSP (r=-0.63, p<0.001), RVEDP (r=-0.55, p=0.001), PASP (r=-0.55, p=0.002), mean PAP (r=-0.51, p=0.003) and LVEDP (r=0.583, p=0.001). Mean septal displacement had the strongest association with hemodynamics compared to the sub-segmental analyses of the basal, mid and apical septum although all were significantly correlated with pressures. In a multivariate regression model with all pressure indices included, RSVP was the only variable that was independently associated with radial displacement of the septum (model R=0.625, p<0.001). While longitudinal displacement of the septum and longitudinal and radial displacement of the RV free wall were all associated with RVEF (r=0.522, p=0.002; r=0.428, p=0.014; r=0.369, p=0038, respectively) they were not sensitive to increased RV preload or afterload.

## Conclusions

The radial displacement of the right sided septal endocardium is inversely related to increased RVSP and may be an useful index detecting early effects of increased afterload on RV mechanics. Conversely, RV free wall displacement is not sensitive to RV afterload despite its association with RVEF.

## Funding

None.

